# Oral contraceptive use and risk of melanoma in premenopausal women

**DOI:** 10.1038/sj.bjc.6690787

**Published:** 1999-11

**Authors:** D Feskanich, D J Hunter, W C Willett, D Spiegelman, M J Stampfer, G A Colditz

**Affiliations:** Channing Laboratory, Department of Medicine, Brigham and Women’s Hospital and Harvard Medical School, 181 Longwood Avenue, Boston, MA 02115, USA; Department of Epidemiology, Harvard Center for Cancer Prevention, Department of Nutrition, Department of Biostatistics andDepartment of Environmental Health, Harvard School of Public Health, Boston, MA 02115, USA

**Keywords:** melanoma, oral contraceptives, oestrogen, premenopausal

## Abstract

Melanoma has been increasing in white populations. Incidence rates rise steeply in women until about age 50, suggesting oestrogen as a possible risk factor. Oestrogens can increase melanocyte count and melanin content and cause hyperpigmentation of the skin. We examined prospectively the association between oral contraceptive (OC) use and diagnoses of superficial spreading and nodular melanoma among 183 693 premenopausal white women in the Nurses’ Health Study (NHS) and the Nurses’ Health Study II (NHS II) cohorts. One hundred and forty six cases were confirmed in NHS during follow-up from 1976 to 1994, and 106 cases were confirmed in NHS II from 1989 to 1995. Skin reaction to sun exposure, sunburn history, mole counts, hair colour, family history of melanoma, parity, height and body mass index were also assessed and included in logistic regression models. A significant twofold increase in risk of melanoma (relative risk (RR) = 2.0, 95% confidence interval (CI) 1.2–3.4) was observed among current OC users compared to never users. Risk was further increased among current users with 10 or more years of use (RR = 3.4, 95% CI 1.7–7.0). Risk did not appear elevated among past OC users, even among those with longer durations of use, and risk did not decline linearly with time since last use. In conclusion, risk of premenopausal melanoma may be increased among women who are current OC users, particularly among those with longer durations of use. Further research is needed to determine whether low-dose oestrogen pills in particular are associated with an increase in risk and to describe possible interactions between OC use and sun exposure or other risk factors for melanoma. © 1999 Cancer Research Campaign


*British Journal of Cancer* (1999) **81**(5), 918–923
© 1999 Cancer Research Campaign
Article no. bjoc.1999.0787

### Oral contraceptive use and risk of melanoma in premenopausal women

**D Feskanich[1], DJ Hunter[1,2,3], WC Willett[1,2,4], D Spiegelman[2,5], MJ Stampfer[1,2] FE Speizer[1,6] and GA Colditz[1,2,3]**

[1]Channing Laboratory, Department of Medicine, Brigham and Women's Hospital and Harvard Medical School, 181 Longwood Avenue, Boston, MA 02115, USA; [2]Department of Epidemiology, [3]Harvard Center for Cancer Prevention, [4]Department of Nutrition, [5]Department of Biostatistics and [6]Department of Environmental Health, Harvard School of Public Health, Boston, MA 02115, USA

**Summary** Melanoma has been increasing in white populations. Incidence rates rise steeply in women until about age 50, suggesting oestrogen as a possible risk factor. Oestrogens can increase melanocyte count and melanin content and cause hyperpigmentation of the skin. We examined prospectively the association between oral contraceptive (OC) use and diagnoses of superficial spreading and nodular melanoma among 183 693 premenopausal white women in the Nurses' Health Study (NHS) and the Nurses' Health Study II (NHS II) cohorts. One hundred and forty six cases were confirmed in NHS during follow-up from 1976 to 1994, and 106 cases were confirmed in NHS II from 1989 to 1995. Skin reaction to sun exposure, sunburn history, mole counts, hair colour, family history of melanoma, parity, height and body mass index were also assessed and included in logistic regression models. A significant twofold increase in risk of melanoma (relative risk (RR) = 2.0, 95% confidence interval (CI) 1.2–3.4) was observed among current OC users compared to never users. Risk was further increased among current users with 10 or more years of use (RR = 3.4, 95% CI 1.7–7.0). Risk did not appear elevated among past OC users, even among those with longer durations of use, and risk did not decline linearly with time since last use. In conclusion, risk of premenopausal melanoma may be increased among women who are current OC users, particularly among those with longer durations of use. Further research is needed to determine whether low-dose oestrogen pills in particular are associated with an increase in risk and to describe possible interactions between OC use and sun exposure or other risk factors for melanoma.

**Keywords:** melanoma; oral contraceptives; oestrogen; premenopausal

Over the last 20 years, the incidence of melanoma has been rising rapidly in white populations in the USA and other parts of the world (Liu and Soong, 1996). Sun exposure, particularly when intermittent (Holman et al, 1986; Osterlind et al, 1988*a*) or when resulting in burns or blisters (Osterlind et al, 1988*a*; MacKie et al, 1989), is a strong risk factor for melanoma, and recent changes in lifestyle that embrace exposure to sunlight may partially account for this rise in incidence.

Oestrogens, either alone or in combination with progesterone, increase the number of melanocytes and their melanin content (Snell and Bischitz, 1960) and can cause hyperpigmentation of the skin (Jelinek, 1970) and thus have been implicated as another possible risk factor in the development of melanoma. Among women, incidence rates rise steeply until about age 50, after which the rate of increase slows (Armstrong and English, 1996), further suggesting a role for oestrogen.

Interest in oral contraceptive use was simulated by two early studies from the same cohort in California which reported elevated risk of melanoma among ever versus never users (Beral et al, 1977; Ramcharan et al, 1981). However, subsequent research has not generally supported this finding (Helmrich et al, 1984; Holman et al, 1984; Gallagher et al, 1985; Osterlind et al, 1988*b*; Zanetti et al, 1990; Hannaford et al, 1991; Palmer et al, 1992; Holly et al, 1995; Westerdahl et al, 1996). Failure to find an association between melanoma and oral contraceptive use may be due to the broad categorization of 'ever users'. A few studies have reported modest elevations in melanoma risk among women with longer durations of use or with longer times since first use (Bain et al, 1982; Holly et al, 1983; Beral et al, 1984; Le et al, 1992). However, previous research has been limited by the predominance of case–control designs that rely on recall of oral contraceptive use. In this investigation, we examined prospectively the risk of melanoma by status, duration, and other measures of oral contraceptive use among premenopausal women in two large cohorts: the Nurses' Health Study (NHS) and the Nurses' Health Study II (NHS II).

### METHODS

The NHS (Colditz et al, 1997) is an ongoing cohort study that was begun in 1976 with 121 700 married female nurses 30–55 years of age and residing in 1 of 11 States across the USA. A second cohort, NHS II, was created in 1989 with 116 671 married and unmarried female nurses 25–42 years of age and residing in 1 of 14 States. Both studies were designed to examine prospectively the effects of oral contraceptive use and other lifestyle factors on chronic diseases, particularly cancers and cardiovascular diseases. Information is collected with biennial mailed questionnaires, and a response rate of at least 90% has been achieved in each follow-up cycle. Deaths are ascertained through the National Death Index.

*Received 15 February 1999*
*Revised 19 April 1999*
*Accepted 19 April 1999*

*Correspondence to:* D Feskanich

**Table 1** Age and age-standardized characteristics of premenopausal Caucasian women in the Nurses' Health Study (NHS) and Nurses' Health Study II (NHS II) cohorts by status of oral contraceptive (OC) use and by duration of use among past and current users

| | Status of OC use | | | Duration of OC use (years) | | |
|---|---|---|---|---|---|---|
| | Never | Past | Current | <5 | 5–9.9 | 10+ |
| NHS[a] | | | | | | |
| Person-years (thousands) | 275.2 | 444.7 | 20.3 | 307.5 | 109.3 | 36.7 |
| Age (years, mean) | 44.4 | 42.4 | 38.2 | 41.9 | 42.3 | 44.3 |
| Height (metres, mean) | 1.64 | 1.64 | 1.64 | 1.64 | 1.64 | 1.64 |
| Body mass index (kg $m^{-2}$, mean) | 24.6 | 24.3 | 23.4 | 23.9 | 24.1 | 23.9 |
| Painful burn or blisters after 2 h of sun exposure during childhood (%) | 14 | 15 | 13 | 15 | 13 | 14 |
| 10+ sunburns over lifetime (%) | 37 | 41 | 38 | 41 | 41 | 40 |
| 3+ moles on left arm (%) | 9 | 10 | 9 | 11 | 11 | 9 |
| Red or blonde hair (%) | 14 | 15 | 16 | 15 | 15 | 16 |
| Parent of sibling with melanoma (%) | 3 | 3 | 2 | 3 | 2 | 2 |
| Nulliparous (%) | 7 | 5 | 5 | 5 | 4 | 7 |
| NHS II[a] | | | | | | |
| Person-years (thousands) | 82.7 | 394.9 | 61.2 | 270.0 | 130.9 | 50.3 |
| Age (years, mean) | 35.7 | 36.7 | 32.2 | 36.2 | 35.6 | 36.3 |
| Height (metres, mean) | 1.65 | 1.65 | 1.65 | 1.65 | 1.65 | 1.65 |
| Body mass index (kg $m^{-2}$, mean) | 24.6 | 24.0 | 23.2 | 24.0 | 23.8 | 23.7 |
| Painful burn or blisters after 2 h of sun exposure during childhood (%) | 10 | 18 | 18 | 18 | 18 | 17 |
| 3+ sunburns during teen years (%) | 25 | 28 | 27 | 27 | 29 | 28 |
| 5+ moles on lower legs (%) | 20 | 22 | 22 | 22 | 22 | 22 |
| Red or blonde hair (%) | 18 | 21 | 22 | 20 | 22 | 23 |
| Parent or sibling with melanoma (%) | 4 | 4 | 4 | 4 | 4 | 4 |
| Nulliparous (%) | 35 | 22 | 39 | 22 | 24 | 39 |

[a]The NHS cohort included 79 571 women who were followed from 1976 until 1994; the NHS II cohort included 104 122 women who were followed from 1989 until 1995.

### Oral contraceptive use

On the baseline questionnaires for both cohorts, participants were asked whether they had ever used oral contraceptives (OC), and if so, to list all periods of use. Subsequent biennial questionnaires asked whether OCs had been used during the previous 2 years and, if so, the number of months of use. From the responses to these questions, a status of OC use (never, past, current) was assigned. We also calculated duration of use, age at first use, and time since first use for past and current users and time since last use for past users. These measures characterized participants in the subsequent 2-year follow-up cycle.

In NHS II, participants identified the exact preparation of their OC pill with the aid of a booklet containing photos of all OCs ever marketed in the USA. From this information, we were able to determine oestrogen dosages. The reproducibility and validity of the OC data were evaluated in a study among 215 randomly selected participants from NHS II (Hunter et al, 1997). The biennial questionnaire responses were compared with data from a subsequent telephone interview that used a structured life events calendar to assist recall of past OC use. Agreement for ever versus never use was 99%, and the correlation for duration of use calculated from the two sources was 0.94. Medical records confirmed the identical or equivalent OC brand in 75% of the reports for which data were obtained.

### Covariate information

In both cohorts, age, parity, and body weight were reassessed at each 2-year follow-up cycle. Height was reported only at baseline.

In NHS, skin reaction after 2 or more hours in the sun during childhood, number of sunburns over the lifetime, natural hair colour at age 21, and family history (parental or sibling) of melanoma were reported in 1982 and number of moles on the left arm was reported in 1986. These measures were used to characterize follow-up from baseline in 1976. Family history was reassessed in 1992.

In NHS II, skin reaction after 2 or more hours in the sun during childhood, number of sunburns between ages 15 and 20, number of moles on the lower legs, and family history (parental or sibling) of melanoma were reported at baseline. Natural hair colour at age 21 was reported in 1991 and was used to characterize follow-up from baseline in 1989.

### Study population and case confirmation

Only caucasian women who were premenopausal and had no history of cancer (except non-melanoma skin cancer) were included in the study populations. Participants were followed from the return date of their baseline questionnaire (mailed June 1976 for NHS and September 1989 for NHS II) until a report of melanoma, other cancer, menopause, death, or end of follow-up (1 June 1994 for NHS and 1 June 1995 for NHS II), whichever occurred first. The NHS study population included 79 571 women and 740 182 person-years of follow-up; NHS II included 104 122 women and 538 807 person-years of follow-up.

Among these women, 260 reported a diagnosis of melanoma on the NHS questionnaires from 1976 to 1994 and 216 reported a diagnosis of melanoma on the NHS II questionnaires from 1989 to 1995. Medical records were obtained for 217 (83%) of the NHS

*© 1999 Cancer Research Campaign* 

**Table 2** Cohort-specific and combined relative risks of invasive melanoma by status and duration of oral contraceptive (OC) use among premenopausal Caucasian women in the Nurses' Health Study (NHS) and the Nurses' Health Study II (NHS II). Never OC use is the reference group for all categories

| | NHS | | | | NHS II | | | | |
|---|---|---|---|---|---|---|---|---|---|
| | Cases | Person-years | RR[1] (95% CI[a]) | RR[2] (95% CI[a]) | Cases | Person-years | RR[1] (95% CI[a]) | RR[2] (95% CI[a]) | Combined RR[2] (95% CI[a]) |
| Never OC use | 50 | 275 217 | 1.0 | 1.0 | 14 | 82 657 | 1.0 | 1.0 | 1.0 |
| Past OC use | 88 | 444 700 | 1.1 (0.8–1.6) | 1.1 (0.7–1.5) | 77 | 394 944 | 1.1 (0.6–2.0) | 1.1 (0.6–2.0) | 1.1 (0.8–1.5) |
| < 5 years | 57 | 302 731 | 1.1 (0.7–1.6) | 1.0 (0.7–1.5) | 41 | 251 029 | 0.9 (0.5–1.7) | 0.9 (0.5–1.7) | 1.0 (0.7–1.4) |
| 5–9.9 years | 23 | 100 561 | 1.3 (0.8–2.1) | 1.2 (0.7–2.1) | 24 | 107 265 | 1.3 (0.7–2.5) | 1.3 (0.6–2.5) | 1.2 (0.8–1.9) |
| 10+ years | 7 | 30 204 | 1.3 (0.6–2.8) | 1.2 (0.6–2.7) | 11 | 32 742 | 1.8[b] (0.8–4.1) | 1.7[b] (0.8–3.7) | 1.4[b] (0.8–2.5) |
| Current OC use | 8 | 20 265 | 2.6 (1.2–5.7) | 2.6 (1.2–5.6) | 15 | 61 205 | 1.7 (0.8–3.6) | 1.6 (0.8–3.3) | 2.0 (1.2–3.4) |
| <10 years | 2 | 13 490 | 0.9 (0.2–4.0) | 0.9 (0.2–3.8) | 7 | 42 636 | 1.3 (0.5–3.2) | 1.2 (0.5–3.0) | 1.0 (0.4–2.8) |
| 10+ years | 6 | 6 563 | 5.3 (2.3–12) | 5.2 (2.2–12) | 8 | 17 581 | 2.7 (1.1–6.5) | 2.4 (1.0–5.7) | 3.4 (1.7–7.0) |

RR[1] = relative risk adjusted for age; RR[2] = relative risk adjusted for age, follow-up cycle, skin reaction after 2 hours of sun exposure during childhood, number of sunburns over lifetime (NHS) or during teenage years (NHS II), number of moles on left arm (NHS) or on lower legs (NHS II), hair colour, family history of melanoma, parity, height, and body mass index. [a]95% confidence interval; [b]$P \leq 0.05$ in test for linear trend over categories of OC use (not including never OC use).

reports and for 156 (72%) of the NHS II reports. Obtainment of medical records did not differ by status of OC use. Among the medical records, 146 (67%) in NHS and 106 (68%) in NHS II were confirmed cases of invasive melanoma. These included superficial spreading (20%), nodular (76%) and unspecified (4%) types. Women who reported a diagnosis of melanoma that was not confirmed by medical record (in situ cancer or other skin conditions) or for whom we could not obtain a medical record were excluded from further follow-up.

### Statistical analyses

The NHS and NHS II cohorts were analysed separately. Incidence rates were estimated for never, past and current OC users within 5-year age groups by dividing the number of melanoma cases by the person-time of follow-up in each of the OC status/age group categories, and relative risks for past and current users were estimated by their incidence rates divided by the rate for never users. For each of the cohorts, pooled logistic regression models within 2-year time increments were used to calculate multivariate relative risks adjusted for all covariates simultaneously (D'Agostino et al, 1990). Age-adjusted and multivariate relative risks were similarly calculated for duration of OC use, age at first use, time since first use and time since last use, using never OC use as the reference category. Tests for trend were performed by entering continuous variables into the multivariate logistic regression models, using the median value within each category. Trend tests did not include the reference category of never users.

Analyses of OC status were stratified by a risk score determined by factors which were associated with significant increases in risk of melanoma, including hair colour, sunburn history and mole count. In NHS II, analyses were also stratified by oestrogen dosage in the OC preparation.

To increase the precision of the risk estimates and obtain a single summary of the results from NHS and NHS II, a random effects model (Dersimonian and Laird, 1986) was used to combine the risk estimates. Data were combined only when there was no significant evidence of heterogeneity between results from the separate cohorts.

### RESULTS

The following factors were associated with a significant increased risk of melanoma: red or blond hair colour, having a parent or sibling with melanoma, ten or more sunburns over the lifetime (NHS) or three or more during the teenage years (NHS II), three or more moles on the left arm (NHS) or five or more on the lower legs (NHS II), painful burning or blistering after 2 or more hours of sun exposure during childhood (NHS), nulliparity (NHS II) and a height of 1.68 metres or more (NHS). However, except for parity and skin reaction to sun exposure during childhood, these melanoma risk factors were not related to status or duration of OC use (Table 1). In NHS II, nulliparous women were more likely to be a current than a past OC user and they were more likely to have used OCs for 10 or more years than for shorter periods. Also in this cohort, painful burning or blistering after 2 or more hours of sun exposure during childhood was least likely among the never OC users. In both cohorts, current OC users were younger than never or past users.

In NHS, the multivariate-adjusted relative risk (RR) of melanoma among current OC users was significantly elevated (RR = 2.6, 95% confidence interval (CI) 1.2–5.6) compared to women who never used OCs (Table 2). Statistical adjustment for all measured covariates in the multivariate model did not notably alter the risk estimate from that produced by the simple age-adjusted model. Current OC users in NHS II also experienced an increased risk of melanoma compared to never users (RR = 1.6, 95% CI 0.8–3.3), though the magnitude was not as great as that observed in NHS (*P*-value test for heterogeneity = 0.4). When results from NHS and NHS II were combined, current OC use was

 *© 1999 Cancer Research Campaign*

**Table 3** Relative risks of invasive melanoma by measures of first and last oral contraceptive (OC) use among premenopausal Caucasian women in the Nurses' Health Study (NHS) and the Nurses' Health Study II (NHS II). Never OC use is the reference group for all categories

| | Combined NHS and NHS II | | | |
|---|---|---|---|---|
| | **Cases** | **Person-years** | **RR[1] (95% CI[a])** | **RR[2] (95% CI[a])** |
| Never OC use | 64 | 357,874 | 1.0 | 1.0 |
| Time since last OC use | | | | |
| < 5 years | 41 | 169,614 | 1.5 (1.0–2.3) | 1.2 (0.7–2.0) |
| 5–9.9 years | 29 | 201,838 | 0.9 (0.6–1.4) | 0.8 (0.5–1.3) |
| 10–14.9 years | 41 | 217,598 | 1.0 (0.6–1.6) | 1.0 (0.7–1.6) |
| 15+ years | 49 | 214,371 | 1.3 (0.7–2.2) | 1.5 (0.9–2.5) |
| Time since first OC use | | | | |
| < 10 years | 14 | 135,409 | 0.8 (0.4–1.4) | 0.7 (0.4–1.4) |
| 10–19.9 years | 118 | 531,747 | 1.2 (0.9–1.7) | 1.1 (0.8–1.5) |
| 20+ years | 54 | 226,442 | 1.4 (0.9–2.3) | 1.2 (0.7–1.9) |
| Age at first OC use | | | | |
| < 20 years | 44 | 192,129 | 1.5 (0.8–2.6) | 1.2 (0.7–2.2) |
| 20–24 years | 86 | 403,115 | 1.4 (0.9–2.0) | 1.2 (0.8–1.8) |
| 25+ years | 56 | 295,939 | 1.1[b] (0.7–1.5) | 1.0 (0.7–1.4) |

RR[1] = relative risk adjusted for age; RR[2] = relative risk adjusted for age, follow-up cycle, skin reaction after 2 hours of sun exposure during childhood, number of sunburns over lifetime (NHS) or during teenage years (NHS II), number of moles on left arm (NHS) or on lower legs (NHS II), hair colour, family history of melanoma, parity, height, body mass index, and duration of OC use. [a]95% confidence interval; [b]$P \leq 0.05$ in test for linear trend over categories of OC use (not including never OC use).

associated with a significant twofold increase in risk (RR = 2.0, 95% CI 1.2–3.4). In both cohorts, there was little increase in risk among past OC users compared to never users. A comparison of ever versus never OC use yielded a combined multivariate-adjusted RR of 1.4 (95% CI 0.8–1.6).

We identified women at high risk of melanoma as those with red or blonde hair colour, a history of sunburns (ten or more over the lifetime in NHS, three or more during teenage years in NHS II), or a substantial number of moles (three or more on the left arm in NHS, five or more on the lower legs in NHS II). Women in the high risk category in NHS and NHS II had a combined RR of 2.4 (95% CI 1.7–3.3) compared to those in the low risk category (i.e. all those not at high risk). We then reassessed the association between OC use and melanoma in analyses stratified by high versus low risk. Among the high-risk women, current OC use was associated with a significantly elevated risk of melanoma (combined RR = 3.1, 95% CI 1.6–5.9), while there was no increase in risk among the low-risk women (combined RR = 1.0, 95% CI 0.2–3.9). However, a test for interaction was not significant ($P = 0.7$).

Analyses of duration of OC use were performed separately for past and current users (Table 2). Compared to never OC users, women in the NHS and NHS II with 10 or more years of current OC use had a combined multivariate-adjusted RR of 3.4 (95% CI 1.7–7.0). The risk estimate was higher in NHS than in NHS II. Though data were sparse for current OC users with shorter durations, there was no indication that risk was increased with fewer than 10 years of use. Among past OC users, 10 or more years of use was not associated with significant increases in risk of melanoma (combined multivariate-adjusted RR = 1.4, 95% CI 0.8–2.5). However, the risk was higher in NHS II (RR = 1.7, 95% CI 0.8–3.7) than in NHS, and there was a significant trend over duration categories among past users.

We speculated that the risk of melanoma associated with current OC use was higher in NHS than in NHS II due to the higher oestrogen dosages that were popular during the earlier years of OC use among the NHS cohort. Unfortunately, we had no data on oestrogen dosage in the NHS cohort. Therefore, we examined risk of melanoma by duration of use of low- (< 35 μg), medium- (35–49 μg) and high- (50+ μg) dose oestrogen pills in the NHS II cohort. Though power was limited, a higher oestrogen dose did not appear to be associated with a higher risk of melanoma. Compared to women who never used the low-dose oestrogen pills, the multivariate-adjusted RR of melanoma among women with 5 or more years of low dose use was 1.4 (95% CI 0.6–3.2). This analysis was adjusted for years of medium-dose and high-dose oestrogen use. Similarly, the multivariate RR of melanoma among women with 5 or more years of high-dose oestrogen use compared to those who never used the high-dose oestrogen pills was 1.6 (95% CI 0.9–3.3).

With a twofold increase in risk of melanoma among current OC users, and little increase in risk among past users, we anticipated that risk would be elevated among past users who recently stopped using OCs and that risk would decline with longer times since last use. However, our data did not support this expectation. After controlling for all covariates plus duration of OC use, the combined RR for fewer than 5 years since last OC use was 1.2 (95% CI 0.7–2.0). Risk was not increased even among past users with fewer than 2 years since last use (combined RR = 0.8, 95% CI 0.4–1.8). The median time since last use was 10 years in NHS and 11 years in NHS II.

We tested the hypothesis that risk of melanoma is increased with earlier exposure to OCs by examining time since first use and age at first use. Though the age-adjusted results were suggestive of an increased risk with earlier OC exposure, the multivariate-adjusted analyses did not support this hypothesis. Combined results from NHS and NHS II yielded an RR of 1.2 (95% CI 0.7–1.9) among women with 20 or more years since first OC use and an RR of 1.2 (95% CI 0.7–2.2) among women who began OC use before 20 years of age compared to women who never used OCs (Table 3). To determine whether risk might be greater with an even earlier age at first use, we examined women who began OC use before age 18 in the NHS II cohort (there were few women and no cases within this category in NHS). However, risk was not elevated in this lower age-at-first-use category (RR = 1.0, 95% CI 0.5–2.3).

*© 1999 Cancer Research Campaign*

### DISCUSSION

In this prospective study among premenopausal caucasian women, current OC use was associated with a significant twofold increase in risk of melanoma. The risk appeared stronger in the NHS cohort than in the NHS II cohort. This could be due to the higher dosages of oestrogen in the OC pills that were available during the early years of the NHS study. Lack of data on the composition of the pills taken by NHS participants prevented us from examining this hypothesis in this cohort. In NHS II, there was no evidence that higher estrogen dosages conferred additional risk, though power to assess this association was limited. We found no appreciable overall excess risk of melanoma among past OC users, suggesting that the effects diminish rapidly after OC use is terminated. Even among the past users with fewer than 2 years since last OC use, there was no evidence of an increase in risk of melanoma. An increase in risk among current OC users and a quickly diminishing risk after quitting is similar to the pattern observed for OC use and breast cancer (Collaborative Group on Hormonal Factors in Breast Cancer) and is consistent with a model of tumour promotion rather than tumour initiation.

The lack of association between ever use of OCs and risk of melanoma in previous studies may be due to a preponderance of past versus current users in the study populations, most of which included women in their post-menopausal years. Few studies examined current OC users separately. Palmer et al (1992) reported a non-significant relative risk of 1.5 for current OC use for cases of non-severe melanoma, and Bain et al (1982) found an increase in risk of similar magnitude among women under 40 years of age, a period when OC use is more common.

In both the NHS and NHS II, only current OC users experienced a significant increased risk of melanoma with longer durations of use. Compared to never OC users, durations of 10 or more years of use were associated with an over threefold increase in risk. Failure of some previous studies to observe an increased risk of melanoma with longer durations of OC use may be attributed to a combined analysis of past and current users and a definition of high duration that included women with only 3 or 5 years of OC use (Holman et al, 1984; Gallagher et al, 1985; Zanetti et al, 1990). Among those studies which examined women with 10 or more years of past or current OC use, four reported relative risks comparable to our findings (Holly et al, 1983; Beral et al, 1984; Hannaford et al, 1991; Le et al, 1992), while others found no increase in risk of melanoma (Helmrich et al, 1984; Holly et al, 1995; Osterlind et al, 1988*b*). However, Holly et al (1995) did report a statistically significant relative risk of 1.7 among women with 5 or more years of OC use and fewer than 15 years since first use, a group which likely included many current OC users.

Some have suggested that OCs may have a stronger effect when used earlier in life and that a long latency period may be required before the appearance of melanoma. Le et al (1992) found that past OC users with 15 or more years since first use had a twofold risk for melanoma compared to never users, while no increase in risk was found among women with shorter latency periods. The relative risk increased to threefold among those who also used OCs for 10 or more years. Holly et al (1983) and Beral et al (1984) also found that risk of melanoma was highest among those with both long durations and latency periods. Our data did not support these findings. Risk of melanoma was not significantly elevated among women who began OC use before the age of 20 or for whom 20 or more years had passed since their first use. Other studies have also reported no association between melanoma and time since first OC use or age at first use (Palmer et al, 1992; Holly et al, 1995). There may be an interaction between duration of use and time since first use, as suggested by earlier studies, but we did not have the power to examine this in our data.

It is biologically plausible that oestrogen may have an effect on the development of melanoma. In animal experiments, oestrogen and oestrogen–progesterone combinations stimulate melanogenesis and cause an increase in both intracellular and extracellular melanin content (Snell and Bischitz, 1960). This is thought to be the underlying reason for the hyperpigmentation associated with OC use (Jelinek, 1970). Hyperpigmentation is most commonly caused by sun exposure which is a strong risk factor for melanoma, particularly with exposures during childhood or early adulthood that result in burns or blisters (Weinstock et al, 1989).

Oestrogen receptors have been found in human melanoma tissue (Fisher et al, 1976; Chaudhuri et al, 1980) but their biological significance is uncertain. Though high-affinity binding has been observed, specificity may be poor (McCarty et al, 1980). Initial case reports suggested that tamoxifen could produce tumour regression in patients with metastatic melanoma (Nesbit et al, 1979; Mirimanoff et al, 1981). However, in larger trials, response rates were low (Telhaug et al, 1982; Wagstaff et al, 1982). More recent clinical investigations indicate that the addition of tamoxifen can increase the response rate to chemotherapy (Cocconi et al, 1992; McClay et al, 1992), though the mechanism may not involve an anti-oestrogenic effect on oestrogen receptors (McClay et al, 1993).

We speculated that OC use by itself may not increase the risk of melanoma but may promote carcinogenesis in women with other significant risk factors. In the NHS and NHS II cohorts, risk for current OC use was threefold among the women defined as high risk and was null among those at low risk, though a test for heterogeneity was not significant. This issue merits examination in other studies.

Our study of OC use and melanoma is one of the few prospective investigations on this topic. Another major advantage of this study was the availability of data on other reproductive and non-reproductive risk factors for melanoma which permitted us to control for possible confounding effects. Though we did have a sunburn history and a measure of susceptibility to sunburn, the study was limited by lack of information on sunbathing habits and the use of sunscreen or other protective measures during periods of OC use. This could confound our results if women who use OCs have higher levels of sun exposure. There is also the possibility that detection bias could explain at least part of the increased risk of melanoma observed among current OC users if they are more likely to get diagnosed due to more frequent physician visits. However, in our cohorts, the percent of women with annual physical exams was high and did not differ by OC use.

In conclusion, current use of oral contraceptives was associated with an increased risk of melanoma in premenopausal Caucasian women, particularly among current users with 10 or more years of use. Since this study began in 1976 when higher oestrogen dose pills were commonly used, further research is needed to determine whether the current lower dose pills are also associated with an increase in melanoma risk.

### ACKNOWLEDGEMENTS

Supported by research grants CA40356, CA50385 from the National Institutes of Health.

*© 1999 Cancer Research Campaign*

**REFERENCES**

Armstrong BK and English DR (1996) Cutaneous malignant melanoma. In: *Cancer Epidemiology and Prevention*, 2nd edn, Schottenfeld D and Fraumeni JF (ed), pp. 1281–1312. Oxford University Press: New York

Bain C, Hennekens CH, Speizer FE, Rosner B, Willett W and Belanger C (1982) Oral contraceptive use and malignant melanoma. *J Natl Cancer Inst* **68**: 537–539

Beral V, Ramcharan S and Faris R (1977) Malignant melanoma and oral contraceptive use among women in California. *Br J Cancer* **36**: 804–809

Beral V, Evans S, Shaw H and Milton G (1984) Oral contraceptive use and malignant melanoma in Australia. *Br J Cancer* **50**: 681–685

Chaudhuri PK, Walker MJ, Briele HA, Beattie CW and Das Gupta TK (1980) Incidence of estrogen receptors in benign nevi and human malignant melanoma. *JAMA* **244**: 791–793

Cocconi G, Bella M, Calabresi F, Tonato M, Canaletti R, Boni C, Buzzi BG, Corgna E, Costa P, Lottici R, Papadia F, Sofra MC and Bacchi M (1992) Treatment of metastatic malignant melanoma with decarbazine plus tamoxifen. *N Engl J Med* **327**: 517–523

Colditz GA, Manson JE and Hankinson SE (1990) The Nurses' Health Study: 20-year contribution to the understanding of health among women. *J Women Health* **6**: 49–62

Collaborative Group on Hormonal Factors in Breast Cancer (1996) Breast cancer and hormonal contraceptives: collaborative reanalysis of individual data on 53 297 women with breast cancer and 100 239 women without breast cancer from 54 epidemiological studies. *Lancet* **347**: 1713–1727

D'Agostino RB, Lee MLT, Belanger AJ, Cupples LA, Anderson K and Kannel WB (1990) Relation of pooled logistic regression to time dependent Cox regression analysis: the Framingham Heart Study. *Stat Med* **9**: 1501–1515

DerSimonian R and Laird N (1986) Meta-analysis in clinical trials. *Controlled Clin Trials* **7**: 177–188

Fisher RI, Heifeld JP and Lippman ME (1976) Oestrogen receptors in human malignant melanoma. *Lancet* **2**: 337–339

Gallagher RP, Elwood JM, Hill GB, Coldman AJ, Threlfall WJ and Spinelli JJ (1985) Reproductive factors, oral contraceptives and risk of malignant melanoma: Western Canada melanoma study. *Br J Cancer* **52**: 901–907

Hannaford PC, Villard-Mackintosh L, Vessey MP and Kay CR (1991) Oral contraceptives and malignant melanoma. *Br J Cancer* **63**: 430–433

Helmrich SP, Rosenberg L, Kaufman DW, Miller DR, Schottenfeld D, Stolley PD and Shapiro S (1984) Lack of an elevated risk of malignant melanoma in relation to oral contraceptive use. *J Natl Cancer Inst* **72**: 617–620

Holly EA, Weiss NS and Liff JM (1983) Cutaneous melanoma in relation to exogenous hormones and reproductive factors. *J Natl Cancer Inst* **70**: 827–831

Holly EA, Cress RD and Ahn DK (1995) Cutaneous melanoma in women: reproductive factors and oral contraceptive use. *Am J Epidemiol* **141**: 943–950

Holman CDJ, Armstrong BK and Heenan PJ (1984) Cutaneous malignant melanoma in women: exogenous sex hormones and reproductive factors. *Br J Cancer* **50**: 673–680

Holman CDJ, Armstrong BK and Heenan PJ (1986) Relationship of cutaneous malignant melanoma to individual sunlight-exposure habits. *J Natl Cancer Inst* **76**: 403–414

Hunter DJ, Manson JE, Colditz GA, Chasan-Taber L, Troy L, Stampfer MJ, Speizer FE and Willett WC (1997) Reproducibility of oral contraceptive histories and validity of hormone composition reported in a cohort of US women. *Contraception* **56**: 373–378

Jelinek JE (1970) Cutaneous side effects of oral contraceptive. *Arch Dermatol* **101**: 181–186

Le MG, Cabanes PA, Desvignes V, Chanteau MF, Mlika N and Avril MF (1992) Oral contraceptive use and risk of cutaneous malignant melanoma in a case-control study of French women. *Cancer Causes Control* **3**: 199–205

Liu T and Soong S-J (1996) Epidemiology of malignant melanoma. *Surgical Clinics N Amer* **76**: 1205–1221

MacKie RM, Freudenberger T and Aitchison TC (1989) Personal risk-factor chart for cutaneous melanoma. *Lancet* **2**: 487–490

McCarty KS Jr, Wortman J, Stowers S, Lubahn DB, McCarty KS Sr and Seigler HF (1980) Sex steroid receptor analysis in human melanoma. *Cancer* **46**: 1463–1470

McClay EF, Mastrangelo MJ, Berd D and Bellet RE (1992) Effective combination chemo/hormonal therapy for malignant melanoma: experience with three consecutive trials. *Int J Cancer* **50**: 553–556

McClay EF, Albright KD, Jones JA, Christian RD and Howell SB (1993) Tamoxifen modulation of cisplatin sensitivity in human malignant melanoma cells. *Cancer Res* **53**: 1571–1576

Mirimanoff RO, Wagenknecht L and Hunziger N (1981) Long-term complete remission of malignant melanoma with tamoxifen. *Lancet* **1**: 1368–1369

Nesbit RA, Woods RL, Tattersall MH, Tox RM, Forbes JF, MacKay IR and Goodyear M (1979) Tamoxifen in malignant melanoma [letter]. *N Engl J Med* **301**: 1241–1242

Osterlind A, Tucker MA, Stone BJ and Jensen OM (1988*a*) The Danish case–control study of cutaneous malignant melanoma. II. Importance of UV-light exposure. *Int J Cancer* **42**: 319–324

Osterlind A, Tucker MA, Stone BJ and Jensen OM (1988*b*) The Danish case–control study of cutaneous malignant melanoma: hormonal and reproductive factors in women. *Int J Cancer* **42**: 821–824

Palmer JR, Rosenberg L, Stron BL, Harlap S, Zauber AG, Warchauer ME and Shapiro S (1992) Oral contraceptive use and risk of cutaneous malignant melanoma. *Cancer Causes Control* **3**: 547–554

Ramcharan S, Pellegrin FA, Ray R and Hsu JP (1981) *The Walnut Creek Contraceptive Drug Study*, Vol. III. NIH Publication No 81–564. US Government Printing Office: Washington, DC

Snell RS and Bischitz PG (1960) The effect of large doses of estrogen and estrogen and progesterone on melanin pigmentation. *J Invest Dermatol* **35**: 73–82

Telhaug R, Klepp O and Bormer O (1982) Phase II study of tamoxifen in patients with metastatic malignant melanoma. *Cancer Treat Rep* **66**: 1437

Wagstaff J, Thatcher N, Rankin E and Crowther D (1982) Tamoxifen in the treatment of metastatic malignant melanoma. *Cancer Treat Rep* **66**: 1771

Weinstock MA, Colditz GA, Willett WC, Stampfer MC, Bronstein BR, Mihm MC and Speizer FE (1989) Non-familial cutaneous melanoma incidence in women associated with sun exposure before 20 years of age. *Pediatrics* **84**: 199–204

Westerdahl J, Olsson H, Masback A, Ingvar C and Johsson N (1996) Risk of malignant melanoma in relation to drug intake, alcohol, smoking, and hormonal factors. *Br J Cancer* **73**: 1126–1131

Zanetti R, Franceschi S, Rosso S, Bidoli E and Colonna S (1990) Cutaneous malignant melanoma in females: the role of hormonal and reproductive factors. *Int J Epidemiol* **19**: 522–526

*© 1999 Cancer Research Campaign*
